# Optimization of High-Pressure Extraction Process of Antioxidant Compounds from *Feteasca regala* Leaves Using Response Surface Methodology

**DOI:** 10.3390/molecules25184209

**Published:** 2020-09-14

**Authors:** Anca Becze, Vanda Liliana Babalau-Fuss, Cerasel Varaticeanu, Cecilia Roman

**Affiliations:** INCDO-INOE 2000, Research Institute for Analytical Instrumentation, ICIA, 400293 Cluj-Napoca, Romania; vanda.fuss@icia.ro (V.L.B.-F.); cerasel.varaticeanu@icia.ro (C.V.); cecilia.roman@icia.ro (C.R.)

**Keywords:** extraction optimization, response surface methodology, CO_2_ extraction, *Feteasca regala* leaves, antioxidant capacity, resveratrol

## Abstract

Circular economy principles are based on the use of by-products from one operation as the raw materials in another. The aim of this work is to obtain extracts with high antioxidant capacity and resveratrol content for the superior capitalization of the biomass of *Feteasca regala* leaves obtained during vineyard horticultural operations in spring. In order to obtain a high-quality extract at an industrial level, an optimal extraction process is needed. Central composite design (CCD) was used for the experiment design, which contained three independent variables: the ratio of extraction solvent to solid matter, temperature (°C) and time (minutes). The evaluation of extracts was done by measuring the total antioxidant capacity of the extracts using photo-chemiluminescent techniques, and the resveratrol content using liquid chromatography. Process optimization was done using response surface methodology (RSM). Minitab software version 17.0 was used for the design of experiments and data analysis. Regression analysis showed that the model predicts 87.5% of the variation for resveratrol and 96% for total antioxidant capacity (TAC). The temperature had the biggest influence on the extraction yield. The optimal operational conditions for the extraction method applied had the following conditions: ratio e/m 2.92; 43.23 °C and 55.4 min. A maximum value of 34,623 µg ascorbic acid equivalent (AAE) /mL total antioxidant capacity and 182.4 µg/mL resveratrol content were obtained when the optimal extraction parameters where used. The values obtained in experiments proved that by using RSM an accurate model can be obtained for extraction of *Feteasca regala* leaves.

## 1. Introduction

*Vitis vinifera* is a perennial woody plant of the genus *Vitis*, family *Vitaceae*, that is widespread in the Mediterranean region, Central Europe and southwest Asia, from Morocco and Spain to southern Germany and eastern to northern Iran. In winemaking, grapes are the raw material for obtaining basic products: wines and juices (maximum 80%). From the remaining part, various by-products are formed (20–30%): pomace, bunches, grape seeds, yeasts, coarse sediments, etc. Their main uses are soil fertilizers, the formation of substrate for the production of biomass and animal feed, and food supplements. The seed oil has various applications in the pharmaceutical, cosmetic, and food industries. A superior valorization of the biomass can be done by extracting high-value compounds and using them as active ingredients in different products [1,2].

The main bioactive constituents of the vine are polyphenolic compounds, a wide category of compounds with different chemical structures, which can be divided into two main classes: flavonoid compounds (flavan-3-oli, flavonols and anthocyanins) and stilbenes. Polyphenols have various biological activities that are mainly attributed to their antioxidant activity [3,4].

The leaves have been used in Indian medicine as an antidote and antidiarrheal, and in Europe for hemorrhoids, varicose veins, or other circulatory disorders, as an antidiarrheal and hemostatic being attributed astringent and hemostatic properties. The chemical composition of the leaves is less studied in the literature, studies have reported the presence of total polyphenols (3337.7 mg gallic acid equivalents of/L), (87.3 mg epicatechin/L) with antioxidant action of 70.32% (determined by the DPPH method) and 10.05 mmol Trolox equivalents/L (determined by the ferric-reducing/antioxidant power (FRAP) method). In the composition of the leaves the presence of organic acids (tartaric, malic, oxalic, fumaric, succinic, citric, glyceric acids), vitamins (C, PP, B, folic acid), carotenoids, waxes, proteins, and salts ((5–7%) calcium and potassium bitartrate, calcium maleate) were also detected [5,6,7,8].

The highest concentration of polyphenols was reported in seeds (~2200 mg/g equivalent of gallic acid), then in the epicarp (skin of the fruit, ~400 mg/g equivalent of gallic acid), in fruit (~25 mg gallic acid equivalent/g) and leaves (~350 mg gallic acid equivalent/g). The type of polyphenols varies from one part of the plant to another, as follows: proanthocyanidins are the major compounds in seeds and epicarp, anthocyanins are found mainly in the epicarp in red grape varieties, flavan-3-ol derivatives. In the case of white grain varieties, flavonoids are found mainly in cords and seeds, being mainly represented by catechins, epicatechins, polymers of procyanidins [9].

Due to the rich content of polyphenols, which are found in all parts of the vine plant, the main action of extracts prepared from this plant is antioxidant action, being very useful for formulating cosmetics that protect the integrity of skin and hair against the effects of oxidative stress [10].

Oxidative stress plays an important role in the process of intrinsic (chronological) and especially extrinsic aging (aging due to excessive sun exposure or photoaging) of the skin. In the case of intrinsic aging, there is a disturbance of the calcium gradient in the epidermis, which acts on the stratum corneum of the cornea and reduces the antioxidant capacity of the cells. In the case of photoaging, oxidative stress causes the overexpression of enzymes that degrade extracellular matrix components and dermal fibers (matrix metalloproteases). Sun exposure also causes oxidation of proteins, especially collagen (glycation phenomenon) and lipid peroxidation increases the risk of skin cancers. [11,12,13,14,15].

Resveratrol has important antioxidant activity, being reported in numerous studies as an active principle that reduces the phenomenon of photoaging [11,12]. Cells pretreated with resveratrol have survived better against UV-B irradiation. Decreasing the production of oxygen free radicals reduces damage oxidative in the DNA of neuronal cells and thus confers action to reduce the risk of cancer associated with excessive exposure to solar radiation [6].

The choice of active substance extraction methods is an important step in the superior valorization of biomass resulted from viticulture operations during springtime in vineyards, as improper methods can lead to impaired active ingredients or low extraction yields. The selection of a method is based on the structural diversity of the desired active principles and their physicochemical properties. Traditional extraction methods for solids have been classified into two groups: solid–liquid extraction (ESL) and micro-solid phase extraction (MEFS). Additionally, modern methods have been described in the literature: assisted extraction ultrasonic assisted extraction (UAE), liquid pressure extraction (PLE), subcritical pressure water extraction (SWE), supercritical fluid extraction (SFE) and microwave-assisted extraction (MAE). Compared to the traditional methods, modern methods usually offer higher extraction yields, shorter processing times, lower solvent consumption, and are easy to transfer to the industrial level [16,17].

Supercritical fluid extraction is a very efficient extraction method that has low costs of operation but extremely high costs of equipment, which makes it unreachable for most wine producers. The purpose of the study is the optimization of parameters for the CO_2_ extraction method which is done at an average pressure of 56 bar, which uses ethanol as cosolvent. The pressure of 56 bar was chosen because is the typical pressure found in a CO_2_ gas tank. Using a much lower pressure than the one used in SFE ensures a lower cost of equipment, while the extracts obtained are still of high quality which is proven by the total antioxidant capacity and resveratrol content. The obtained extracts are meant to be used in the cosmetic industry as active ingredients in mouth wash and toothpaste, that is why the use of solvents is restricted.

Many factors influence the extraction. Because the pressure is constant and the use of solvents is limited, the main factors that influence the extraction are solvent to biomass ratio, extraction temperature, and time. The optimization process can be done in two ways: empirical and statistical. In the empirical method, each factor is usually tested and optimized one at a time, which makes the whole proceeding very time consuming and ignores the interaction between the factors. Response surface methodology (RSM) was used to evaluate the effects of different variables on the outcome which is the total antioxidant capacity. Using the RSM statistical method ensures a less time-consuming process and an evaluation of the interactions among different factors [18,19].

The response surface method (RSM) examines the relationship between some input variables and one or more output variables. The method was first introduced in 1951 by G.E.P Box and K. B. Wilson. RSM provides optimization with the help of polynomials adapted to the data obtained from optimization procedure designed experiments [19,20,21,22].

In the response surface method, although second-order polynomials are generally used to model complex systems, higher-order polynomials can also be used (Equation (1)) [19]:

(1) $$Y=\beta_{0}+\overset{k}{\underset{i=1}{\sum }}\beta_{i}X_{i}+\overset{k}{\underset{i<j}{\sum }}\beta_{ij}X_{i}X_{j}+\overset{k}{\underset{i=1}{\sum }}\beta_{ii}X_{i}^{2}+\cdots$$

where β_0_, β_i_, β_ij_, and β_ii_ are the regression coefficients for the intercept, linear, quadratic and interaction terms, respectively, and X_i_ and X_j_ are the independent variables.

The creation of the response surface model is performed by estimating the β coefficients shown above with the collected data. Estimation of these coefficients is possible with the least-squares regression [19].

## 2. Results and Discussion

The results obtained after all 20 experiments were performed are presented in Table 1.

The results from the response surface regression: resveratrol versus ratio e/m, temperature, time are presented in Table 2.

The total degrees of freedom (DF) is the amount of information in pure data. The analysis uses the information to estimate the values of unknown population parameters. Adjusted sums of squares are measures of variation for different components of the model and measure how much variation a term or a model has. To calculate the *p*-value for a term and the adjusted R2, statistic Minitab software uses the adjusted mean squares. Usually, the *p*-values and the adjusted R2 statistic are interpreted instead of the adjusted mean squares. The F-value is the statistic test used to determine whether the term is associated with the response, and it is used to calculate the *p*-value. The *p*-value is a probability that measures the evidence against the null hypothesis, which is used to decide the statistical significance of the terms and model. A sufficiently large F-value indicates that the term or model is significant. The *p*-value is large (*p*-value > 0.05) for the ratio of solvent to matter and time which proves that they have a low significance to the regression model as predicted by central composite design (CCD). The temperature has a low *p*-value (*p*-value < 0.05) which indicates a very high significance for the regression model [23].

**Figure 1:**
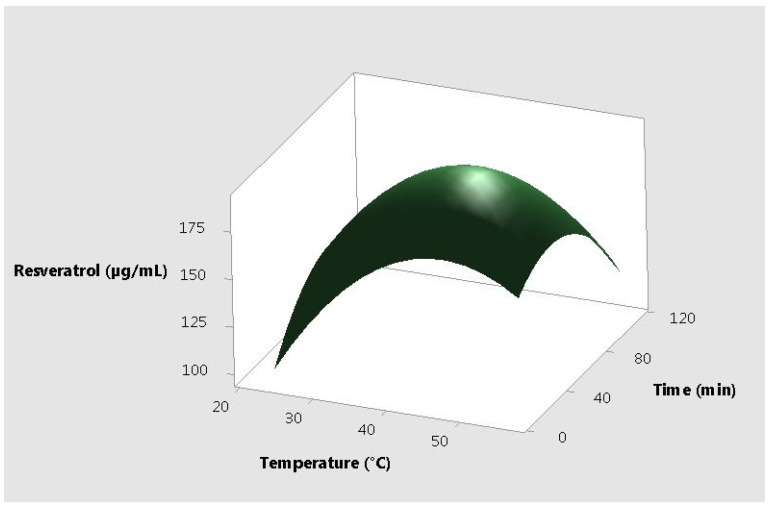
Surface plot resveratrol vs. time and temperature.

**Figure 2:**
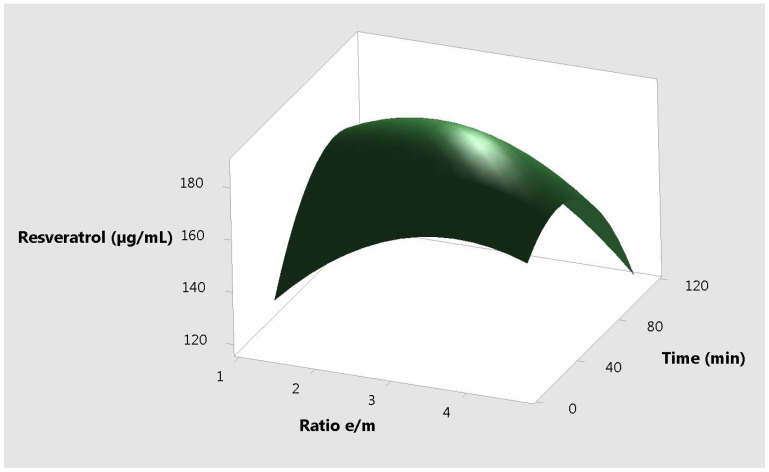
Surface plot resveratrol vs. time and ratio e/m.

**Figure 3:**
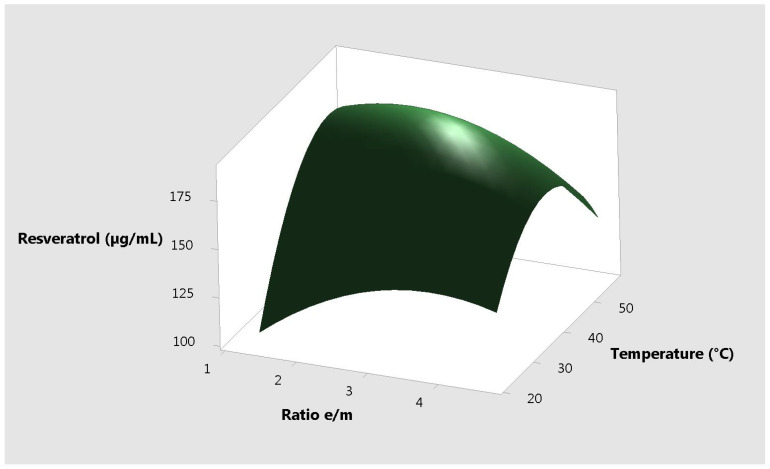
Surface plot resveratrol vs. temperature and ratio e/m.

The response plots for resveratrol vs. the 3 different input variables, correlated two by two are presented in Figure 1, Figure 2 and Figure 3.

The response surface regression: TAC versus ratio e/m, temperature and time, obtained from de data using Minitab, is presented in Table 3.

In the case of the total antioxidant capacity the *p*-value is large (*p*-value > 0.05) for the ratio of extraction solution to matter, which proves that they have a low significance to the regression model as predicted by CCD. Time and temperature have a low *p*-value (*p*-value < 0.05), which indicates an extremely high significance for the regression model.

**Figure 4:**
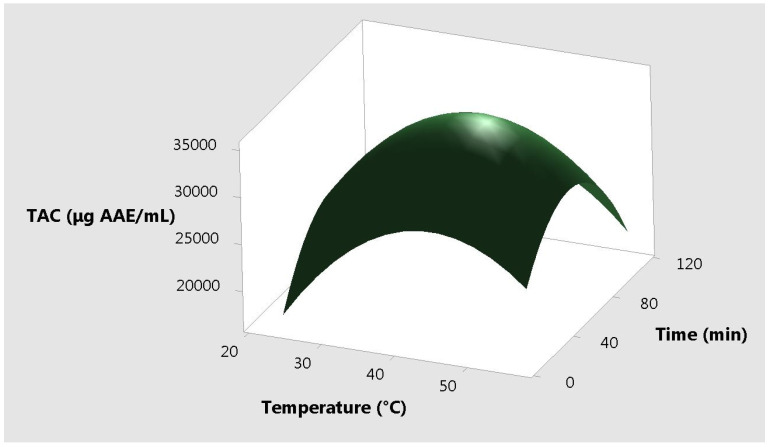
Surface plot of the total antioxidant capacity vs. time and temperature.

**Figure 5:**
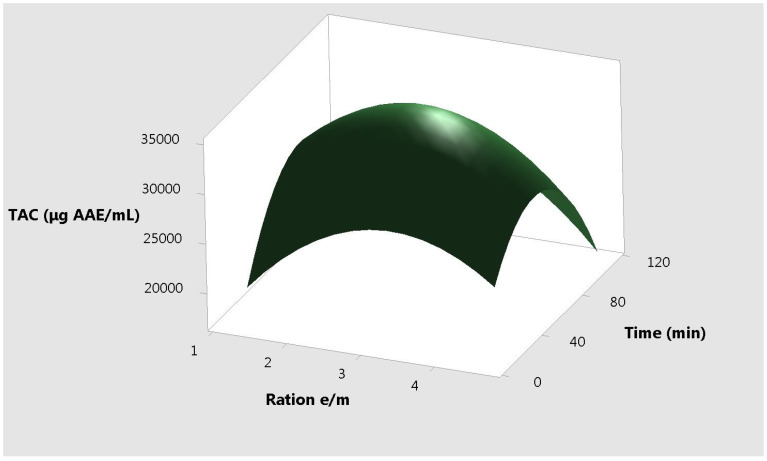
Surface plot of the total antioxidant capacity vs. time and ratio e/m.

**Figure 6:**
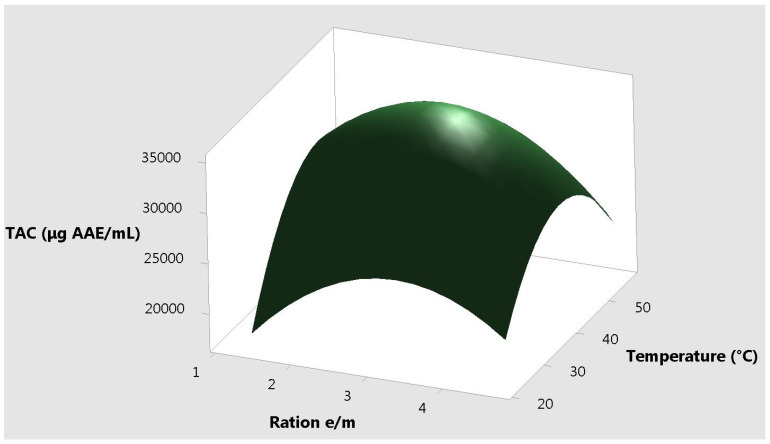
Surface plot of the total antioxidant capacity vs. temperature and ratio e/m.

The response plots for resveratrol vs. the three different input variables, correlated two by two are presented in Figure 4, Figure 5 and Figure 6. It can be observed that the extremities of these variables are useless for the extraction yield.

The analysis of the response surface model for resveratrol content and the total antioxidant capacity was verified by measuring the R2 coefficient (Table 4).

Adjusted R2 is the percentage of the variation in the response that is explained by the model, adjusted for the number of predictors in the model relative to the number of observations. Adjusted R2 is calculated as 1 minus the ratio of the mean square error (MSE) to the mean square total (MS Total).

Predicted R2 is calculated with a formula that is equivalent to systematically removing each observation from the data set, estimating the regression equation, and determining how well the model predicts the removed observation. The value of predicted R2 ranges between 0% and 100%. The low R-sq pred value (29.01%) indicates that the model for resveratrol content is over-fit. An over-fit model occurs when terms for effects are added that are not important in the population. That is not the case for the total antioxidant capacity where the R-sq (pred) is 70.71%.

The response equation in unceded unites obtained for resveratrol content and total antioxidant capacity is presented in Equations (2) and (3):

(2) $$\begin{array}{c} \mathrm{Resveratrol}=-286.6+69.4\cdot X_{1}+14.21\cdot X_{2}+2.415\cdot X_{3}-5.85\cdot X_{1}\cdot X_{1}-0.1320\cdot X_{2}\cdot X_{2}\\ \;-0.01058\cdot X_{3}\cdot X_{3}-0.589\cdot X_{1}\cdot X_{2}-0.195\cdot X_{1}\cdot X_{3}-0.0167\cdot X_{2}\cdot X_{3} \end{array}$$

(3) $$\begin{array}{c} \mathrm{TAC}=-57367+16425\cdot X_{1}+2542\cdot X_{2}+486.4\cdot X_{3}-2050\cdot X_{1}\cdot X_{1}-26.55\cdot X_{2}\cdot X_{2}\\ \;-3.139\cdot X_{3}\cdot X_{3}-67.1\cdot X_{1}\cdot X_{2}-26.1\cdot X_{1}\cdot X_{3}-1.33\cdot X_{2}\cdot X_{1} \end{array}$$

where:

X_1_—Ratio e/m

X_2_—Temperature

X_3_—Time

The model indicated that the linear effects of temperature and time had the greatest significance on total antioxidant capacity.

The data was used to select the optimal process parameters in order to obtain the highest concentration of resveratrol and total antioxidant capacity. The parameters for the optimized extraction process are presented in Table 5.

Composite desirability assesses how well a combination of variables satisfies the goals that have been defined for the responses. It is calculated as the weighted geometric mean of the individual desirability using the Formula (4) [24]:

(4) $$D={\left(d_{1}\cdot d_{2}\cdot \ldots \cdot d_{n}\right)}^{\frac{1}{n}}$$

where:

D—Composite desirability

d*_n_*—Individual desirability for the *n*th response

Desirability has a range of 0 (zero) to 1 (one). 1 (one) represents the ideal case; 0 (zero) indicates that one or more responses are outside their acceptable limits. In the case of the experiment, the composite desirability has a value very close to 1, i.e., 0.95466 which indicates that the parameters have a positive result for all responses as a whole.

### Verification of Experiments

A comparison of predicted and experimental values for the response variables resveratrol content and total antioxidant activity is presented in Table 6. The parameters used for the experiments are ratio e/m 3, temperature 43 °C, and the time 56 min. The extraction was done 3 times with the same parameters to ensure that the results have repeatability. The standard deviation was calculated for the obtained results.

The observed values were lower than the predicted one, but with 0.61% for TAC and 3.5% for the resveratrol content, which represent very low differences. The experimental results prove that model obtained has a high accuracy rate of prediction.

The optimal process parameters obtained using RSM is similar to the optimal regime obtained by Silva et al. [24] but very different from the optimal method developed by Majeed et al. [25] and Parveen who’s optimal regime is 16 h extraction time and 1:20 ratio of material to extraction solvent. These can be explained by the extraction method used and the material extracted [24,25,26]. CO_2_ was specifically chosen to ensure that very good extraction yield is obtained in a short time. The RSM can be used to optimize other extraction method for very different biomass so no valuable compounds found in them are lost [27,28].

Angelov et al. [29] have studied the extraction of resveratrol from grapevine stems using a mixture of ethanol and water as the solvent [29]. They concluded that a higher ration of solvent to solid ratio, 5:1 is better for the extraction. The difference between the ratio obtained in this research, 3:1, and the one Angelov et al. [29], 5:1, can be explained based on the water content of the biomass used for the extortion. Leaves used in the current study have a much higher water content than grapevine stems.

Matloub [30] performed a 30 min extraction at room temperature from *Vitis vinifera* leaves with different solvent mixtures. The results proved that hydro-ethanolic extracts had the highest antioxidant activity [30]. Nabli et al. [31], optimized the extraction of anthocyanin from Grenache noir (*Vitis vinifera* L.) vine leaf compering the SO_2_ extraction with the ethanol extraction, both using water as co-solvent. A much shorter time was needed for the ethanol extraction, 3–4.37 h, compared to 5.77–6 h for SO_2_ extraction [31]. Each of the results obtained from the different studies align with the results obtained in this study which prove that a shorter time and better recovery can be obtained when using ethanol as solvent.

## 3. Materials and Methods

### 3.1. Chemicals

Ethanol was bought from Merck and the ultra-pure water was obtained using EVAPUR by Siemens water purification system. ACW kit was purchased from Analitik Jena.

The leaves and vines used for the extraction resulted from horticultural work applied to vines in spring. They were provided by company Jidvei, as biomass resulted in their 2019 spring horticultural work on their *Feteasca regala* vineyard located in Alba County, Romania. The samples were stored at −20 °C until the extraction.

### 3.2. Extraction Process

Priviest studies proved that the high-pressure CO_2_ extraction with ethanol as co-solvent resulted in a total antioxidant capacity 20–25 times higher than a regular extraction using just ethanol.

High-pressure CO_2_ extraction was performed using a Parr Instruments 1-L benchtop reactor with a 4875 Power Controller. The power controller ensures the monitoring of the pressure and the temperature as well as the continuous agitation of the sample. Ethanol was chosen as co-solvent because of its molecular structure which makes possible the extraction of both polar and non-polar substances and because the boiling point of ethanol is 78.1 °C under normal atmospheric conditions which makes ethanol easy to be removed from extracts. The concentrated extracts can be used as active ingredients in the cosmetics industry, for obtaining mouth wash and toothpaste without heaving any negative impact due to the extraction solvent.

The experimental design started with the evaluation of the *Feteasca regala* plant by-products to be extracted. The ratio between the dry matter (leaves and vines) and the co-solvent (ethanol) must ensure homogeneity and fluidity so that the stirring elements in the extractor don’t get blocked. The homogeneity of the mixture of biomass and ethanol must be done with adequate equipment to ensure that the mixture does not heat up and does not suffer any degradation due to the heat. CO_2_ extraction can be performed at different temperature and pressure levels. The mixture of ethanol and leaves at different ratios (ration e/m 2:1; 3:1, 4:1) were blended at high speed in a laboratory-grade blender for 5 min. The volume of the solution introduced in the Parr extractor was always 600 mL no matter the ratio of plant to co-solvent. At 600 mL solution volume the consumption of CO_2_ is 34.8 L measured under normal conditions (pressure of 1 atm and temperature of 0 °C) for each extraction process. Depending on the experimental design different time and temperature were set. The speed of the stirrer was set the same for all experiments, 1000 rpm. After the extraction was finished the mixture of ethanol and leaves was removed from the Parr extractor and then centrifuged at 3500 rpm for 10 min. The supernatant was then collected and stored in amber glass bottles at 4–5 °C.

### 3.3. Determination of Total Antioxidant Capacity

The determination of the total antioxidant capacity was performed after diluting the sample to be analyzed with ultra-pure water, followed by analysis by Photochem Analytik Jena, Germany which combines the photochemical excitation of free radicals with luminometric detection. The results were expressed in ascorbic acid equivalents (AAE). An ACW Kit was used for the analysis, which consists of a dilution solution for water-soluble samples, buffer solution, photosensitizer solution, standard antioxidant solution.

### 3.4. Determination of Resveratrol Content

To determine the resveratrol content of the extracts, a high-performance liquid chromatograph (HPLC) Series 200 from Perkin Elmer (Shelton, CT, USA) was used, with a UV detector and data processing system. The chromatographic separation was performed on an Agilent (SB-C18 150 mm 3.5 µm × 0.3 mm, Santa Clara, CA, USA) column at 30 °C. Chromatographic analysis was conducted in an isocratic mode. The mobile phase consisted of a mixture of methanol and ultrapure water (80:20, *v*/*v*) at a constant flow rate of 1 mL/min. The detection was carried out at 306 nm. An injection volume of 10 μL was used for all standards and samples.

### 3.5. Experimental Design and Statistical Analysis

Because the products will be used in the cosmetic industry, there is a limited number of solvents that can be used for the extraction. Based on preliminary experiments, ethanol was chosen as a co-extraction solvent and a range of temperature, time and the ratio of ethanol to material was established. Minitab software, version 17, was used for statistical calculations and analysis of data. Central composite design (CCD) was used for the design of experiments, with three input variables: the ratio of ethanol to raw material (X1), temperature (X2, °C), time (X3, min) and two output variables: antioxidant capacity (µg equiv. AAE /mL) and resveratrol (µg/mL).). In order to simplify the data ratio of extraction solvent to raw material was expressed not as fraction, 2:1, but as a number 2. The range of independent variables and their levels are presented in Table 7, which was based on the results of preliminary experiments.

Table 8 presents each experiment that was performed. The data contains the design of 20 experimental points and 6 replicates. The effects of unexplained variability in the observed response due to extraneous factors were minimized by randomizing. The order of experiments at the center of the design was used for the estimation of a pure error sum of squares.

### 3.6. Model Verification

Optimal conditions to obtain high antioxidant capacity and high levels of resveratrol content from *Feteasca regala* leaves were obtained using the predictive equations of RSM. The extraction was repeated 3 times with the same parameters. The total antioxidant activity and resveratrol content were determined for all 3 extracts obtained under optimal conditions. The results obtained from the experiment and predicted values were compared in order to determine the validity of the model.

## 4. Conclusions

The optimal parameters for the extraction process resulted from the response surface analysis are: ratio of extraction solvent to raw material 3:1, temperature 43 °C and extraction time 56 min. Based on the data obtained, a prediction model was established with a higher correlation to the total antioxidant capacity and to the resveratrol content. The experiments conducted for the verification prove accuracy of the model and the optimization of the extraction process.

The obtained extracts have a very high antioxidant capacity which proves not only the high value of leaves, that are considered waste, as raw material in obtained the extracts but also the efficiency of the optimized extraction process.

The optimized method is fast and efficient for the extraction of resveratrol and other compounds with antioxidant capacity from *Feteasca regala* leaves and can be applied for the extraction of leaves from other grapes varieties.

## Figures and Tables

**Table 1:** Experimental results.

Ratio of Ethanol to Plant Material	Temperature (°C)	Time (min)	Total Antioxidant Capacity (µg AAE /mL)	Resveratrol Content (µg/mL)
4	30.00	90.00	25,331	152.1
3	40.00	60.00	34,793	190.1
2	50.00	30.00	28,719	172.4
3	40.00	60.00	34,726	188.5
2	30.00	90.00	26,397	158.7
3	40.00	60.00	33,978	175.3
3	40.00	110.45	25,408	155.2
2	30.00	30.00	24,947	141.8
4	50.00	90.00	24,825	139.1
3	40.00	19.55	28,819	167.8
3	40.00	60.00	34,896	181.5
3	23.18	60.00	24,097	124.6
4	30.00	30.00	26,781	160.8
4.5	40.00	60.00	29,937	174.4
3	56.82	60.00	31,093	177.6
1.2	40.00	60.00	28,674	169.4
4	50.00	30.00	28,103	165.7
3	40.00	60.00	34,394	194.9
2	50.00	90.00	28,805	167.1
3	40.00	60.00	34,735	190.5

**Table 2:** Analysis of variance for resveratrol content.

Source	Degrees of Freedom (DF)	Adjusted Sums of Squares (Adj SS)	Adjusted Mean Squares (Adj MS)	F-Value	*p*-Value
Model	9	5675.70	630.63	7.77	0.002
Linear	3	1216.72	405.57	5.00	0.023
Ratio e/m	1	14.13	14.13	0.17	0.685
Temperature	1	1055.03	1055.03	13.00	0.005
Time	1	147.56	147.56	1.82	0.207
Square	3	3705.73	1235.24	15.23	0.000
Ratio e/m ∗ Ratio e/m	1	493.00	493.00	6.08	0.033
Temperature ∗ Temperature	1	2512.07	2512.07	30.96	0.000
Time ∗ Time	1	1307.69	1307.69	16.12	0.002
2-Way Interaction	3	753.25	251.08	3.09	0.076
Ratio e/m ∗ Temperature	1	277.30	277.30	3.42	0.094
Ratio e/m ∗ Time	1	274.95	274.95	3.39	0.095
Temperature ∗ Time	1	201.00	201.00	2.48	0.147
Error	10	811.32	81.13		
Lack-of-Fit	5	557.90	111.58	2.20	0.203
Pure Error	5	253.42	50.68		
Total	19	6487.02			

**Table 3:** Analysis of variance for total antioxidant capacity (TAC).

Source	DF	Adj SS	Adj MS	F-Value	*p*-Value
Model	9	274,182,082	30,464,676	26.49	0.000
Linear	3	31,824,976	10,608,325	9.22	0.003
Ratio e/m	1	212,586	212,586	0.18	0.676
Temperature	1	25,775,042	25,775,042	22.41	0.001
Time	1	5,837,348	5,837,348	5.08	0.048
Square	3	232,582,223	77,527,408	67.42	0.000
Ratio e/m ∗ Ratio e/m	1	60,576,745	60,576,745	52.68	0.000
Temperature ∗ Temperature	1	101,583,872	101,583,872	88.34	0.000
Time ∗ Time	1	115,028,536	115,028,536	100.03	0.000
2-Way Interaction	3	9,774,882	3,258,294	2.83	0.092
Ratio e/m ∗ Temperature	1	3,596,562	3,596,562	3.13	0.107
Ratio e/m ∗ Time	1	4,904,712	4,904,712	4.27	0.066
Temperature ∗ Time	1	1,273,608	1,273,608	1.11	0.317
Error	10	11,499,604	1,149,960		
Lack-of-Fit	5	10,912,332	2,182,466	18.58	0.003
Pure Error	5	587,272	117,454		
Total	19	285,681,686			

**Table 4:** Model Summary.

	S	R-sq	R-sq (ad)	R-sq (pred)
Resveratrol content	9.00733	87.49%	76.24%	29.01%
Total antioxidant capacity	1072.36	95.97%	92.35%	70.71%

**Table 5:** Response optimization: TAC, resveratrol solution.

Ratio e/m	Temperature (°C)	Time (min)	Expected Value for Total Antioxidant Capacity (µg AAE /mL)	Expected Value for Resveratrol Content (µg/mL)	Composite Desirability
2.92	43.2	55.4	34,840	189	0.955

**Table 6:** Results obtained for optimal parameter.

Parameter	Predicted Value	Observed Value
Total antioxidant capacity (µg AAE /mL)	34,839	34,623 ± 29.7
Resveratrol content (µg/mL)	189.0	182.4 ± 15.3

**Table 7:** Independent variables and the factor levels of the experiment.

Independent Variables	Factor Levels		
	−1	0	1
Ratio (e/m)	2	3	4
Temperature (°C)	30	40	50
Time (min)	30	60	90

**Table 8:** Design of experiments.

Standard Order	Run Order	Ratio e/m	Temperature (°C)	Time (min)
6	1	4	30.00	90.00
19	2	3	40.00	60.00
3	3	2	50.00	30.00
15	4	3	40.00	60.00
5	5	2	30.00	90.00
18	6	3	40.00	60.00
14	7	3	40.00	110.45
1	8	2	30.00	30.00
8	9	4	50.00	90.00
13	10	3	40.00	30.00
16	11	3	40.00	60.00
11	12	3	23.18	60.00
2	13	4	30.00	30.00
10	14	4.5	40.00	60.00
12	15	3	56.82	60.00
9	16	1.2	40.00	60.00
4	17	4	50.00	30.00
20	18	3	40.00	60.00
7	19	2	50.00	90.00
17	20	3	40.00	60.00

## References

[1] Aradhya M., Dangl G., Prins B., Boursiquot J., Walker M., Meredith C., Simon C. (2003). Genetic structure and differentiation in cultivated grape, *Vitis vinifera* L.. Genet. Res..

[2] Tartaglione L., Gambuti A., De Cicco P., Ercolano G., Ianaro A., Tagliatela-Scafati O., Moio L., Forino M. (2018). NMR-based phytochemical analysis of *Vitis vinifera* cv *Falanghina* leaves. Characterization of a previously undescribed biflavonoid with antiproliferative activity. Fitoterapia.

[3] Fraternale D., Rudov A., Prattichizzo F., Olivieri F., Ricci D., Giacomini E., Carloni S., Azzolini C., Gordillo B., Jara-Palacios M.J. (2016). Chemical composition and “*in vitro*” anti-inflammatory activity of *Vitis vinifera* L. (var. *Sangiovese*) tendrils extract. J. Funct. Foods.

[4] Barba F.J., Zhu Z., Koubaa M., SantAna A.S., Orlien O. (2016). Green alternative methods for the extraction of antioxidant bioactive compounds from winery wastes and by-products: A review. Trends Food Sci. Technol..

[5] Xia E.Q., Deng G.F., Guo Y.J., Li H.B. (2010). Biological Activities of Polyphenols from Grapes. Int. J. Mol. Sci..

[6] Fiume M.M. (2012). Safety Assessment of Vitis vinifera (Grape) Ingredients as Used in Cosmetics.

[7] Katalinic V., Generalic I., Skroza D., Ljubenkov I., Teskera A., Konta I., Boban M. (2009). Insight in the phenolic composition and antioxidative properties of *Vitis vinifera* leaves extracts. Croat. J. Food Sci. Technol..

[8] Dresch R.R., Dresch M.K., Guerreiro A.F., Biegelmeyer R., Holzschuh M.H., Rambo D.F., Henriques A.T. (2014). Phenolic Compounds from the Leaves of *Vitis labrusca* and *Vitis vinifera* L. as a Source of Waste Byproducts: Development and Validation of LC Method and Antichemotactic Activity. Food Anal. Methods.

[9] Sousa E.C., Uchôa-Thomaz A.M.A., Carioca J.O.B., de Morais S.M., de Lima A., Martins C.G., Alexandrino C.D., Ferreira P.A.T., Rodrigues A.L.M., Rodrigues S.P. (2014). Chemical composition and bioactive compounds of grape pomace (*Vitis vinifera* L.), Benitaka variety, grown in the semiarid region of Northeast Brazil. Food Sci. Technol..

[10] Nunes M.A., Rodriguez F., Oliveira M.B., Galanakis C.M. (2017). Grape Processing By-Products as Active Ingredients for Cosmetic Proposes. Handbook of Grape Processing By-Products.

[11] Rinnerthaler M., Bischof J., Streubel M.K., Trost A., Richter K. (2015). Oxidative Stress in Aging Human Skin. Biomolecules.

[12] Crişan D., Crişan M., Moldovan M., Lupşor M., Badea R. (2012). Ultrasonografic assessement of the cutaneous changes induced by topical flavonoid therapy. Clin. Cosmet. Investig. Dermatol..

[13] Schnee S., Queiroz E.F., Voinesco F., Marcourt L., Dubuis P.H., Wolfender J.L., Gindro K. (2013). *Vitis vinifera* canes, a new source of antifungal compounds against *Plasmopara viticola*, *Erysiphe necator*, and *Botrytis cinerea*. J. Agric. Food Chem..

[14] Cornacchione S., Sadick N.S., Neveu M., Talbourdet S., Lazou K., Viron C., Renimel I., de Quéral D., Kurfurst R., Schnebert S. (2007). In vivo skin antioxidant effect of a new combination based on a specific *Vitis vinifera* shoot extract and a biotechnological extract. J. Drugs Dermatol..

[15] Teixeira A., Baenas N., Dominguez-Perles R., Barros A., Rosa E., Moreno D.A., Garcia-Viguera C. (2014). Natural bioactive compounds from winery by-products as health promoters: A review. Int. J. Mol. Sci..

[16] Yahya N.A., Attan N., Wahab R.A. (2018). An overview of cosmeceutically relevant plant extracts and strategies for extraction of plant-based bioactive compounds. Food Bioprod. Process..

[17] Bimakr M., Russly A.R., Taip F.S., Ganjloo A., Salleh L.M., Selamat J., Hamid A., Zaidul I.S.M. (2011). Comparison of different extraction methods for the extraction of major bioactive flavonoid compounds from spearmint (*Mentha spicata* L.) leaves. Food Bioprod. Process..

[18] Park N.Y., Lee G.D., Jeong Y.J., Kwon J.H. (1998). Optimization of extraction conditions for physicochemical properties of ethanol extracts from *Chrysanthemum boreale*. Korean J. Food Nutr..

[19] Liyana-Pathirana C., Shahidi F. (2005). Optimization of extraction of phenolic compounds from wheat using response surface methodology. Food Chem..

[20] Gan C.Y., Latiff A.A. (2011). Optimisation of the solvent extraction of bioactive compounds from *Parkia speciosa* pod using response surface methodology. Food Chem..

[21] Kwak H.J., Park S.J., Kim Y.N., Yoo G., Jeong E.J., Kim S. (2019). Optimization of extraction conditions for enhancing estrogenic activity of *Rheum undulatum* Linne’ using response surface methodology. Sep. Sci. Technol..

[22] Kwon J.H., Belanger J.M.R., Pare J.R.J. (2003). Optimization of microwave-assisted extraction (MAP) for ginseng components by response surface methodology. J. Agric. Food Chem..

[23] Minitab. https://support.minitab.com/en-us/minitab-express/1/help-and-how-to/modeling-statistics/anova/how-to/two-way-anova/interpret-the-results/all-statistics-and-graphs/.

[24] Silva E.M., Rogez H., Larondelle Y. (2007). Optimization of extraction of phenolics from *Inga edulis* leaves using response surface methodology. Sep. Purif. Technol..

[25] Majeed M., Hussain A.I., Chatha S.A.S., Khosa M.K.K., Kamal G.M., Kamal M.A., Zhang X., Liu M. (2016). Optimization protocol for the extraction of antioxidant components from *Origanum vulgare* leaves using response surface methodology. Saudi J. Biol. Sci..

[26] Parveen K.G., Santosh K.V., Sharma A.K. (2018). Optimization of extraction protocol of *Parmelia perlata* and its validation for protective effects against oxalate-induced renal injury in NRK-52E cells. J. Herb. Med..

[27] Wu Y., Cui S.W., Tang J., Gu X. (2007). Optimization of extraction process of crude polysaccharides from boat-fruited sterculia seeds by response surface methodology. Food Chem..

[28] Lee W.C., Yusof S., Hamid N.S.A., Baharin B.S. (2006). Optimizing conditions for enzymatic clarification of banana juice using response surface methodology (RSM). J. Food Eng..

[29] Angelov G., Boyadzhiev L., Georgieva S. (2016). Useful Bioactive Substances from Wastes: Recovery of Trans-Resveratrol from Grapevine Stems. Open Chem. Eng. J..

[30] Matloub A. (2018). Optimization of polyphenol extraction from *Vitis vinifera* L. leaves, antioxidant activity and its correlation with amelioration effect on AlCl3-induced Alzheimer’s disease. Arch. Pharm. Sci. Ain Shams Univ..

[31] Nabli R., Achour S., Jourdes M., Teissedre P.-L., Helal A.N., Ezzili B. (2012). Anthocyanin composition and extraction from Grenache noir (*Vitis vinifera* L.) vine leaf using an experimental design. I-By ethanol or sulfur dioxide. J. Int. Sci. Vigne Vin.

